# Tissue Proteomics and Custom-Made Transcriptomics Analysis Tool Revel PPP1R12B, FBLN5, CRIP2, and POSTN as Potential Prognostic Biomarkers in Prostate Cancer

**DOI:** 10.1200/GO.22.62000

**Published:** 2022-05-05

**Authors:** Juan Bizzotto, Rosario Lavignolle, Agustina Sabater, Pablo Sanchis, Sofia Lage-Vickers, Rocio Seniuk, Gaston Pascual, Pia Valacco, Elba Vazquez, Javier Cotignola, Geraldine Gueron

**Affiliations:** ^1^ CONICET (IQUIBICEN)—Universidad de Buenos Aires, Buenos Aires, Argentina

## Abstract

Prostate cancer (PCa) is a progressive disease involving multiple molecular alterations. The Gleason Grade (GG) is used as an aid for physicians to evaluate the prognosis of men with PCa using samples from prostate tissues. However, some PCa that are histo-pathologically grouped in the same GGs can differ significantly in outcome. The aim of this study was to identify molecular biomarkers that could behave independently from GG and other clinical-pathological variables (age, GG and TMPRSS2-ERG fusion status) that can improve risk prediction of PCa.

**METHODS:**

An in-depth proteomics analysis (LC/ESI-MS/MS) was performed on human prostate adenocarcinoma and benign prostate hyperplasia (BPH) tissues. Protein candidates enriched in PCa vs BPH were further evaluated in-silico using a custom-made pipeline. For this purpose, seven publicly available transcriptomic PCa datasets (ntotal = 875) were selected, and differential expression analyses were performed in R. A Shiny-based tool was then built to execute the search and to allow visualization across multiple datasets. Further multivariable analyses were performed using clinical-pathological parameters as covariates.

**RESULTS:**

Proteomics analysis yielded a list of 89 proteins enriched in PCa compared with BPH. 58 showed high expression in PCa datasets and nine of those, showed significant association with higher risk of death. Further, when performing a multivariable Cox proportional-hazard model including age, GG and TMPRSS2-ERG fusion status, PPP1R12B, FBLN5, CRIP2, and POSTN displayed associations with poor prognosis, independently from the other co-variates (HR = 1.5, *P* = .0084; HR = 1.34, *P* = .035; HR = 1.36, *P* = .016 and HR = 1.4, *P* = .0028, respectively).

**CONCLUSION:**

PPP1R12B, FBLN5, CRIP2, and POSTN proteins were enriched in PCa human tissues. Our custom pipeline revealed increased expression of these genes across multiple PCa datasets and significant association with increased risk of death, independent from known risk factors and predictors of PCa prognosis. Thus, these factors rise as potential prognostic markers.

